# Health Tract, No. 137

**Published:** 1865

**Authors:** 


### HEALTH TRACT, No. 137.

### PHILOSOPHY.

PRACTICAL philosophy is that which enables us to look at the ills of life, its disappointments and its diseases, in a manner which does much to surmount them and deprive them of the power to do any permanent injury. True philosophy has no pretense about it; no chicanery, no fraud; it does not worry itself in the endeavor to make the worse appear the better reason, or in making troublesome concealments; on the contrary, it finds a happiness and a grateful relief even in a frankness which endangers a storm of ridicule. Who, for example, does not admire the moral courage of the elderly negro noticed upon the hurricane-deck of a steamer, after the taking of Fort Donelson; with a philosophical and retrospective cast of countenance, he squatted down on his little bundle, toasting himself against the chimney, in a state of most profound meditation.

"Were you in the fight?"

"Had a little taste of it, sa."

"Stood your ground, did you?"

"No, sa, I runs."

"Run at the first fire, did you?"

"Yes, sa, and would hab run soona, had I knowd it war coming."

"Why, that was not very creditable to your courage."

"Dat isn't in my line, sa—cookin's my perfeshun."

"Well, but have you no regard for your reputation?"

"Reputation's nuffin to me by de side ob life."

"Do you consider your life worth more than other people's?"

"It's worth more to me, sa."

"Then you must value it very highly?"

"Yes, sa, I does—more dan all dis wuld—more dan a million ob dollars, sa; for what would dat be worth to a man wid de bref out ob him? Self-preserbashun am de first law wid me."

"Then patriotism and honor are nothing to you?"

"Nuffin whatever, sa—I regard dem as among de vanities."

There is another kind of philosophy, or which may be called a moral force, which often enables men to live above disease, and survive for many years, ravages on the constitution, which, preying upon persons of less strength of mind, would hurry them to the grave in a very short time. We remember to have heard of a neighbor in early youth named Hume. He was a great miser and very rich. He was apparently at the point of death. All his broad and fertile acres had been disposed of, and he ceased to dictate to his lawyer, who, knowing he had a large amount of silver and gold in his house, said to him after a pause: "Well, Mr. Hume, what disposition will you make of your money?" "My money! do you expect me to give away my money, too! I will not do it;" and summoning to himself what, under the circumstances, seemed to be a superhuman energy, he rose from his bed, dressed himself, broke the spell of his disease, and lived some years afterward to advocate the making of tin hats, as they would not soon wear out.

Of two persons having consumption, with apparently equal chances of life, the man who abandons himself to his fate, hugs the fire, and is afraid to stir out of doors lest he should take cold, inevitably dies in a short time; the other, having force of character, indomitable determination, and a truer philosophy, considers that life is worth striving for, that he can but die any how, and braving all winds and weathers, fights courageously against his malady, and lives to be an old man. So it is in some forms of paralysis, rheumatism, and other disablements, the exercise of a true philosophy is manifested in brave resolves to live down disease, to live above it, and by sheer force of will to break the spell which was thrown over the succumbing body; thus the mind may, and often does become a power over human maladies more efficient than the most famed medicines of the apothecary.

